# Impact of Aerosol Dust on xMAP Multiplex Detection of Different Class Pathogens

**DOI:** 10.3389/fmicb.2017.02341

**Published:** 2017-11-29

**Authors:** Denis A. Kleymenov, Vladimir A. Gushchin, Alexander L. Gintsburg, Artem P. Tkachuk

**Affiliations:** ^1^Translational Biomedicine Laboratory, N.F. Gamaleya Federal Research Centre for Epidemiology and Microbiology, Moscow, Russia; ^2^Department of Virology, Faculty of Biology, Moscow State University, Moscow, Russia; ^3^N.F. Gamaleya Federal Research Centre for Epidemiology and Microbiology, Moscow, Russia

**Keywords:** multiplex detection, xMAP, Luminex, aiRDeTeX, RNA virus, DNA virus, gram-negative bacteria, toxin

## Abstract

Environmental or city-scale bioaerosol surveillance can provide additional value for biodefense and public health. Efficient bioaerosol monitoring should rely on multiplex systems capable of detecting a wide range of biologically hazardous components potentially present in air (bacteria, viruses, toxins and allergens). xMAP technology from Luminex^TM^ allows multiplex bead-based detection of antigens or nucleic acids, but its use for simultaneous detection of different classes of pathogens (bacteria, virus, toxin) is questionable. Another problem is the detection of pathogens in complex matrices, e.g., in the presence of dust. In the this research, we developed the model xMAP multiplex test-system aiRDeTeX 1.0, which enables detection of influenza A virus, Adenovirus type 6 *Salmonella typhimurium*, and cholera toxin B subunit representing RNA virus, DNA virus, gram-negative bacteria and toxin respectively as model organisms of biologically hazardous components potentially present in or spreadable through the air. We have extensively studied the effect of matrix solution (PBS, distilled water), environmental dust and ultrasound treatment for monoplex and multiplex detection efficiency of individual targets. All targets were efficiently detectable in PBS and in the presence of dust. Ultrasound does not improve the detection except for bacterial LPS.

## Introduction

Bioaerosol is a known source of biologically hazardous components or pathogenic biological agents (PBAs). Bioaerosol might contain pathogenic bacteria, viruses, toxins and allergens (Macher et al., 2012). Aerosol-transmitted infectious agents cause the greatest concern for natural epidemic cases (Makarov et al., 2017) and as a source of potential bioterrorism (Barras and Greub, 2014). The overall list of bioaerosol threats includes more than 40 PBAs (Centers for Disease Control and Prevention, 2017). However, no commercially available test-system for efficient multiplex bioaerosol surveillance is implemented to reduce the risk of aerosol infections in modern cities.

One of the best-known multiplex diagnostic platforms is the Multiple Analyte Profiling technology (xMAP; Luminex Corp., Austin, TX, United States). The idea behind this technology is 40 years old (Fulwyler, 1976; McHugh, 1994) and suggests the use of suspended polystyrene microbeads as a biosensor surface instead of a microplate bottom. Multiplexity is provided by a color code of different bead populations. The variation of two or three dyes concentrations inside the individual beads is used to create panels of 50, 100, and 500 differently coded regions ready to be combined in a single multiplex. The magnetic properties of Luminex beads enable easy sample preparation in manual and automatic modes (Dzenitis and Makarewicz, 2010). Uncomplicated surface chemistry allows straightforward custom coupling of any molecular targets. Altogether, Luminex bead multiplex immune assay (MIA) technology has found many applications in areas of fundamental and applied diagnostic studies (Reslova et al., 2017).

xMAP-based technology is compatible with different kinds of immunodetection techniques of any molecular targets, which makes the Luminex technology particularly useful for multiplex detection of pathogen surface targets with different characteristics. Luminex technology has the capability to differentiate a surface protein target (e.g., adenovirus hexon), a surface non-protein/lipid + carbohydrate target (e.g., LPS), a single protein target (toxin), and internal protein target (e.g., influenza virus nucleocapsid). Indirect serological detection of antibodies against protozoa (the causative agent of malaria), viruses (the causative agent of Ebola hemorrhagic fever), and bacteria (the causative agent of leptospirosis) has been described in the last 2 years in the scientific literature (Kerkhof et al., 2015; Wynwood et al., 2015; Ayouba et al., 2017). A competitive type of immunoassay is less represented (Czeh et al., 2012; Guo et al., 2013).

xMAP technology has been repeatedly tested for the detection of different PBAs that cause intestinal infections in water and food, toxins and potential bioterrorism agents. The detection of *Brucella* spp. *O*-antigen lipopolysaccharide by in-house monoclonal antibodies (mAbs) showed impressive sensitivity with the limit of detection (LOD) from 2 × 10^2^ to 8 × 10^4^ cells/ml depending on the type of Brucella (Silbereisen et al., 2015). The same authors studied the possibility of multiplex detection of different strains of anthrax spores with an LOD from 10^3^ to 10^4^ spores/ml (Tamborrini et al., 2010). Another study performed multiplex detection of bacterial pathogens (*Escherichia coli* O157: H7, *Salmonella typhimurium, Campylobacter jejuni*, and *Listeria monocytogenes*) and toxin [staphylococcal enterotoxin B (SEB)] in food with an LOD from 10^2^ to 10^6^ CFU/ml for bacteria and 0.064 ng/mL for the toxin. Importantly, this study described the effect of different food samples as a matrix (e.g., chicken, spinach, or milk) on the efficacy of PBA detection compared to PBS (Kim et al., 2010).

Multiplex detection of various isolates of the virus of salmon infectious anemia (ISAV) is an important example of an application of the xMAP immunoassay for biosurveillance purposes (Hoare et al., 2016). An assay developed using the nucleoprotein mAbs appears to be a rapid and sensitive method for detecting and quantifying ISAV. This assay has the potential to be multiplexed for the detection of other fish pathogens with LOD from 1/5000 to 1/1000 dilutions of the virus-containing sample at a concentration of 2.2 × 10^6^ tissue culture infective dose (TCID)_50_/ml.

Research on the xMAP MIA detection of various toxins is quite widespread in the scientific literature. Importantly immunoassay is the only way to detect toxins as they are small molecules, peptides or proteins not containing nucleic acids assessable by other methods. Garber et al. (2010) showed multiplex detection of abrin, botulinum toxin A, ricin and SEB with LOD from 0.01 to 1.3 μg/ml in different food matrices. Fruit juices, chocolate milk, cola, baby food and PBS were used as matrices (Garber et al., 2010). In another study, the possibility of multiplex immunodetection of SEA, cholera toxin, botulinum toxin A and ricin (LOD was 0.01 ng/ml) as well as SEB and labile toxin of *E. coli* (LT) (0.1 ng/ml) with PBS-BSA as matrix was demonstrated. Milk used as a matrix led to a significant sensitivity decrease. LOD decreased 2- to 5-fold for most toxins and 30-fold for LT (Simonova et al., 2012). This research demonstrates the importance of investigating the matrix effect during multiplex immune assay development.

Ultrasound (US) disintegration is widely used in the study of microorganisms and particularly bacteria. US treatment is used for the disintegration of bacteria during the extraction of DNA, proteins or components of cell wall. It was shown that US disintegration could be integrated as sample preparation module in flow through bioaerosol surveillance systems (Dzenitis and Makarewicz, 2010). Value of US treatment for manual preparation of environmental samples has not been explicitly studied in context of MIA detection.

The purpose of the present study was to develop the model xMAP bioaerosol multiplex immunoassay aiRDeTeX 1.0 [RNA virus (influenza A virus), DNA virus (Adenovirus 6), gram-negative bacteria (*Salmonella* spp.) and toxin (cholera toxin B) — version 1] — test-system and to determine the parameters of individual PBA detection in various matrices. Particularly, the effects of fine dust and US treatment for pathogen detection in pure water and PBS in single- and multiplex regimens for each target were extensively studied. This study describes for the first time the successful multiplex immunodetection of bioaerosol pathogens from four biological classes (RNA virus, DNA virus, bacteria and toxin).

## Materials and Methods

### Antibodies and Antigens

The study used four PBA imitators and eight antibody preparations — two Ab clones for each target. RNA-virus detection monoplex was against internal nucleoprotein (NP). As the target imitator, influenza A virus (IAV), subtype H5N2 [NCBI, KX879578-85] (Smirnov et al., 2000), kindly provided by Dr. M. Shmarov, was used. For capture and detection, mouse monoclonal antibodies to the nucleocapsid protein of the avian influenza virus clones NP3 and NPS, respectively, obtained by Dr. A. Yu. Kozlov and kindly provided by Prof. T. V. Grebennikova, were used. The clone of NPS was conjugated to biotin isothiocyanate using EZ-Link^TM^ Sulfo-NHS-Biotin kit (Thermo Fisher, #21326) according to the manufacturer’s protocol.

DNA-containing virus detection monoplex was designed against surface protein (hexon) antigen from Adenovirus type 6 (AdV6). Strain Tonsil 99 (Bialexa, Russia) preparation was used as the model antigen. Mouse monoclonal antibodies to AdV6 hexon (clone SY-25), kindly provided by Prof. T. V. Grebennikova, were used as the capture antibody. Clone 1AD mouse monoclonal antibodies to AdV6 hexon (Bialexa, Russia) conjugated with biotin was used as a detection antibody.

As a non-pathogenic bacteria imitator, *Salmonella enterica* serovar *typhimurium* MvP728 (STm), prepared from a strain of wild-type *S. typhimurium* NCTC12023 kindly provided by Dr. V. G. Lunin, was used. Virulence was suppressed in these strain by a double deletion dHtrA/dPurD. Clone ST1 mABs (Bialexa, Russia or 1E6, Abcam) against LPS *S. typhimurium* was used as capture antibody. The same biotinylated aliquot was used as detection antibody.

Cholera toxin B-subunit (CTB) (Sigma, #C9903) was used as a toxin imitator. The preparation CTB mABs (clone CT8) and CTB mABs (clone CT9) preparations conjugated with biotin (Bialexa, Russia) were used as the primary and detecting antibodies, respectively.

### Matrices and Spiked Samples

Pathogenic biological agent, potentially present in bioaerosol, are typically collected and transferred into a liquid medium before analysis. At the same time, the fine-dispersed dust, which will inevitably penetrate the sample in the process of collecting the desired targets, is a side effect of the analysis. As matrices for the analysis, we compared four media using combination of SASS^®^ 4000 Aerosol Concentrator attached to SASS^TM^ 2300 Wetted-Wall Air Sampler (Research International, United States). We used 5 min of aerosol collection in an open forest park on a sunny, windy summer day at a temperature of 20°C – 25°C for collection of the ambient air contents (1–10 microns size dust in accordance with the characteristics of the device) in PBS pH 7.4 (Amresco, #E404), or in deionized water type 1, 18.2 MOm. These two types of media are named here as PBS-DUST and H2O-DUST. Initial media without dust PBS and H2O as a control were used through the study. Five minutes intervals was enough to pass through 15 000 L of aerosol.

Mono- and multi-spiked samples were prepared by adding PBAs to each of the four matrices of a certain concentration and then by double dilution six times (seven concentrations in total). To extract the internal target — nucleoprotein — from the virion of the influenza A virus, 0.1% Igepal CA-630 [Sigma, #I3021] was added to the matrices before the start of the assay.

During the initial studies, a wide range of target concentrations was analyzed. The range of concentrations of each PBA for presentation in the paper was determined as follows: the lowest concentration in the multiplex at which the MFI values of PBA in all four matrices are higher than the LOD. In addition to this concentration, six sequential doubly increased concentrations were included for each PBA. Finally the following PBA concentrations were analyzed: IAV in concentration from 4000 to 63 ng/ml (about 1.5^∗^10^10^ to 1^∗^10^8^ virions/ml); AdV6 in concentration from 2000 to 32 ng/ml (about 8^∗^10^9^ to 1^∗^10^8^ virions/ml); STm from 10^7^ to 10^1^ CFU/ml, then the most informative range of concentrations were chosen ranging from 10^6^ to 1.5^∗^10^4^ CFU/ml and CTB from 128 to 2 ng/ml.

### xMAP Analysis

Immunoassay was performed using xMAP technology from Luminex. Primary ABs in concentration 10 μg/10^6^ microspheres was conjugated with 4 regions: NP3 (anti-NP IAV) with no.15, SY-25 (anti-hexon AdV6) with no.45, ST1 (anti-LPS STm) with no.72 and CT8 (anti-CTB) with no.78. The microsphere coupling was carried out by carbodiimide chemistry coupling protocol in accordance with the protocol given by Luminex (2016). The solutions necessary for the coupling of microspheres were prepared using the following reagents: NaH_2_PO_4_⋅H_2_O (Helicon, #Am-O823) for activation buffer (0.1 M NaH_2_PO_4_, pH 6.2); MES hydrate [Sigma, #M2933] for coupling buffer (50 mM MES, pH 5.0), and 1-ethyl-1-3-dimethylaminopropyl-carbodiimide hydrochloride (EDC) (Sigma, #22980) and *N*-hydroxysulfosuccinimide (s-NHS) (Sigma, #24520). The coupling procedure is described briefly as follows: 1 × 10^6^ beads were activated with 10 μl of 50 mg/mL EDC and 10 μl of 50 mg/mL s-NHS in 80 μl activation buffer for 20 min at 25°C with gentle mixing by vortex at 10 min intervals. After that, the activated beads were washed two times, and resuspended in 500 μL of coupling buffer with the addition of 10 μg/10^6^ microspheres coupling Abs. After incubation for 2 h with mixing (by 20 rpm rotation) at room temperature in the dark and 3 washing steps, the microspheres were resuspended in 1 ml of blocking/storage buffer with minimum overnight storing before analysis. The microspheres remaining after the coupling procedure were counted using an automatic cell counter TC-20 (Bio-RAD, United States).

As a microsphere blocking/storage buffer, PBS-TBN (PBS, 0.1% BSA, 0.02% Tween-20, 0.05% NaN3) for no.15-NP3 and no.78-CT8 was used. PBS-BN (PBS, 1% BSA, 0.05% NaN3) was used for no.45-SY-25. Pierce^TM^ Protein-Free (PBS) Blocking Buffer (Pierce, #37584) for no.72-ST1 was used. To prepare PBS-TBN and PBS-BN, BSA (Sigma, #B-4287), Tween 20 [Helicon, #Am-O777], and NaN3 [Helicon, #Am-O639] were used.

Immunological analysis was carried out according to the manufacturer’s instructions (Luminex, 2016). Briefly, in a 96-well polistirol flat-bottom plate, [Greiner, #GR-655001] 50 μl PBS-TBN with 2500 microspheres of each region and 50 μl of one of four spiked matrices were added. Shaking-incubation conditions were as follows: 1 h, +37°C and 800 rpm. Further washing included two cycles of adding and mixing on the shaker (30 s 800 rpm) and removal of 100 μl of PBS-TBN in each well. The same washing conditions were applied for all washing steps. Spheres were resuspended in 50 μl of PBS-TBN. Detecting antibodies at a concentration of 8 μg/ml in 50 μl PBS-TBN were used. The mixture was incubated on a shaker for 1 h (37°C and 800 rpm) and then washed. Before the third incubation, 50 μl of PBS-TBN for bead resuspension was added followed by 50 μl of SAPE solution (Thermo, #S866) at a concentration of 8 μg/ml in PBS-TBN. The third incubation was performed for 30 min at +25°C and 800 rpm, followed by the final washing step and resuspension in 100 μl of PBS-TBN. The sample was analyzed in the MagPix analyzer (Luminex, United States). It was considered sufficient to have at least 100 or more microspheres of each region per well.

### Ultrasonic Treatment

For the disintegration of two multi-spiked matrices PBS-DUST and H_2_O-DUST with all four PBAs (IAV, AdV6, STm and CTB in concentrations of 1000 ng/ml, 500 ng/ml, 2.5^∗^10^5^ CFU/ml and 32 ng/ml, respectively) in the device for laboratory ultrasound studies of the Volna-L [630 W, 22 ± 1.65 kHz] series, Model UZTA-0,63/22-OL manufactured by the Center of Ultrasonic Technologies LLC (Biysk, Russia) was used. The procedure was carried out using a cup horn for the indirect sonication mode. According to the manufacturer, the intensity of ultrasonic exposure for this method of use is from 3 to 10 W/cm^2^, which is regulated by the output power controller from 30 to 100%, respectively, with linear dependence.

Three samples of each matrix in a volume of 4 ml each, in triplicates, at the level of 30, 65, and 100% power for 15 min were sonicated. At time intervals of 5 s, 30 s, 1, 2, 5, 10, and 15 min, 70 μl of sample was removed from the sonicator. For comparison, samples for each run similar in composition but not exposed to ultrasound were analyzed.

### Statistical Analysis

The results of the study were processed with parametric statistical methods using the statistical program GraphPad Prism 6 (GraphPad Software, Inc., United States). The type of data representation and the methods of statistical processing are described in figure legend. LODs were determined as the lowest concentration tested with a signal greater than the mean background fluorescence plus three times the standard deviation. The fluorescent threshold (FT) line indicator depending on the matrix for each target was slightly different. In view of that heterogeneity, each mean value of the PBA of every matrix in mono- and multiplex was normalized relative to each other by subtracting the corresponding FT value. All measures were assayed in three replicates.

## Results

### Initial Optimizations

The immunological xMAP multiplex test system development underwent an extensive optimization process. The amount of capture antibodies (2, 5, 10, 20 μg per 1 million microspheres), time for the first two of the three incubations (30–60 min), incubation temperature (+25°C or +37°C), with or without shaking, concentration of detecting antibodies (4, 6, or 8 μg/ml), concentration of SAPE (4, 6, or 8 μg/ml) and the number of washes (2–4 cycles) were optimized for each particular target (**Supplementary Figures S1, S2**). Various blocking reagents (PBS-TBN, PBS-BN and Pierce^TM^ Protein-Free (PBS) Blocking Buffer) were used to reduce the non-specific background and improve specificity. SuperBlock^TM^ (PBS) (Thermo, #37580), Blocker^TM^ casein in PBS (Thermo, #37582) and gelatin from the skin of cold water fish (Sigma, #G7765) were also tested, but none of these were subsequently used because of the lack of blocking effect (**Supplementary Figure S3**).

For the influenza virus A NP, an optimization of the lysing reagent was also performed. *N*-lauroylsarcosine sodium salt (NLS) (Helicon, #Am-O719), ethylenediaminetetraacetic acid (EDTA) (Helicon, #Am-O105B), sodium deoxycholate (Sigma, #D6750) and Igepal CA-630 (Sigma, #I3021) were tested. Earlier, the lysis capacity of NLS was tested for two concentrations of NLS (0.1 and 0.5%) (Leirs et al., 2016). All four detergents in concentration 0.1% were tested in this study (**Supplementary Figure S4**). The best results were shown for 0.1% Igepal CA-630, both for prior lysis and for lysis combined with Ab-coupled microspheres incubation. There was no significant effect of 0.1% Igepal CA-630 on the results of the other PBA multiplex detection. Therefore, 0.1% Igepal CA-630 was used for the initial incubation buffer conditions.

### Singleplex xMAP Detection

#### RNA Virus Detection

**FIGURE 1:**
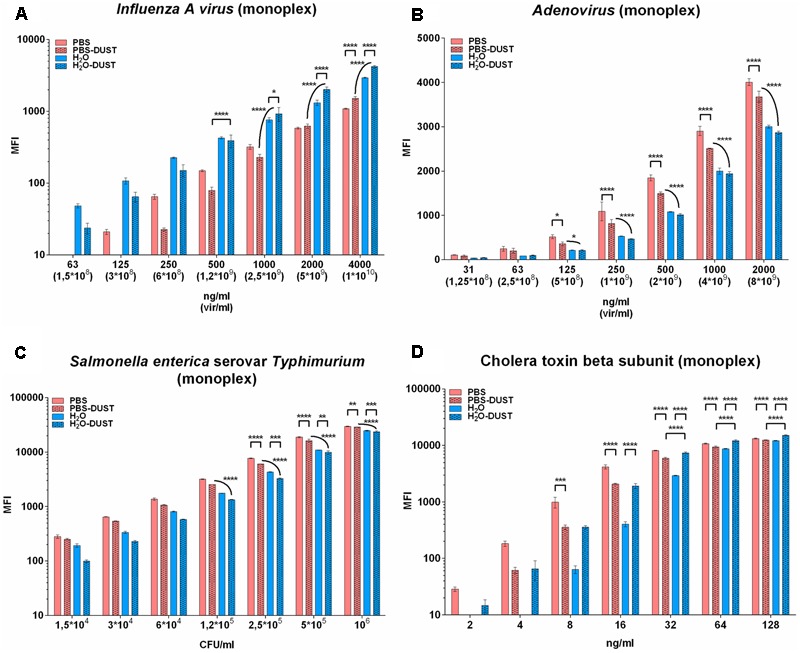
The results of PBA detection in monoplex in different matrices. Results for influenza A virus subtype H5N2 **(A)**, adenovirus type 6 strain Tonsil 99 **(B)**, *S. typhimurium* **(C)** and CTB **(D)** are shown. The data are presented as the mean of three replicates, from which one standard deviation is postponed. Each mean value of the PBA of every matrix was normalized relative to each other by subtracting the corresponding LOD value. Statistical analysis was carried out by the method of multiple comparisons using the Tukey criterion. The only significance between PBS against PBS-DUST, H_2_O against H_2_O-DUST and PBS-DUST against H_2_O-DUST are shown. Other significance bars are hidden for convenience. Statistical significance is: ^∗^*p* < 0.05; ^∗∗^*p* < 0.01; ^∗∗∗^*p* < 0.001; ^∗∗∗∗^*p* < 0.0001. The groups being compared are indicated by the endings of arcs and staples.

As the model for RNA virus detection, influenza A virus subtype H5N2 was used. Internal target NP was detectable with FT above 24–37 MFI (depending on type of buffer conditions). It is known that an internal target is more resistant to antigenic variations; thus, it could provide reliable detection of different influenza A subtypes. The LOD was in line with at least 125 ng/ml in PBS and at least 63 ng/ml in water corresponding to 10^8^ viral particles per 1 ml. The presence of dust in both PBS and water decreased the MFI in low NP concentrations but increased the MFI for concentrations higher than 2000 ng/ml for PBS and 1000 ng/ml for water (**Figure 1A**).

#### DNA Virus Detection

As a model for DNA virus detection adenovirus type 6, strain Tonsil 99 was used. Surface protein AdV6 hexon (AdVH) was detectable above FT from 97 to 100 MFI (depending on the type of buffer conditions) corresponding to 10^8^ viral particles per 1 ml of solution. The highest MFI values for AdVH among different matrices were found to be those obtained with PBS. These results were significantly higher than those in the presence of dust or in water conditions (**Figure 1B**).

#### Bacteria Detection

As a model for bacteria detection, gram-negative *S. typhimurium* was used. LPS detection at different bacterial concentrations ranging from 10^7^ to 10^1^ CFU/ml were tested (**Supplementary Figure S5**). Positive results above FT at 22–49 MFI (depending on the buffer conditions) determined an LOD of the monoplex assay that varied from 10^3^ CFU/ml for water or both PBS matrices to 10^4^ CFU/ml for water in the presence of dust. For further research, a range of concentrations from 10^6^ to 1.5 ^∗^ 10^4^ CFU/ml was used (**Figure 1C**). The best results for the detection of *S. typhimurium* LPS was achieved in PBS. Dust slightly decreased the MFI for both water and PBS significantly from 10^5^ CFU/ml and higher.

#### Toxin Detection

As a model for toxin detection, CTB was used. CTB was detectable with a fluorescent threshold above 82–91 MFI (depending on the type of buffer conditions) (**Figure 1D**). The best results for the detection of CTB were achieved in PBS. Dust significantly decreased the MFI for PBS but significantly improved detection in water. The sensitivity of CTB monoplex was 2 ng/ml.

#### Multiplex xMAP Detection

After studying the analytical properties of a singleplex assay, the multiplexing of selected targets was optimized. For multiplex panel development, four types of individually conjugated beads were combined at ratios of 1:1:1:1 (2500 beads each per well). To determine the FT of the multiplex test system, different matrices without antigens were tested in triplicate.

**FIGURE 2:**
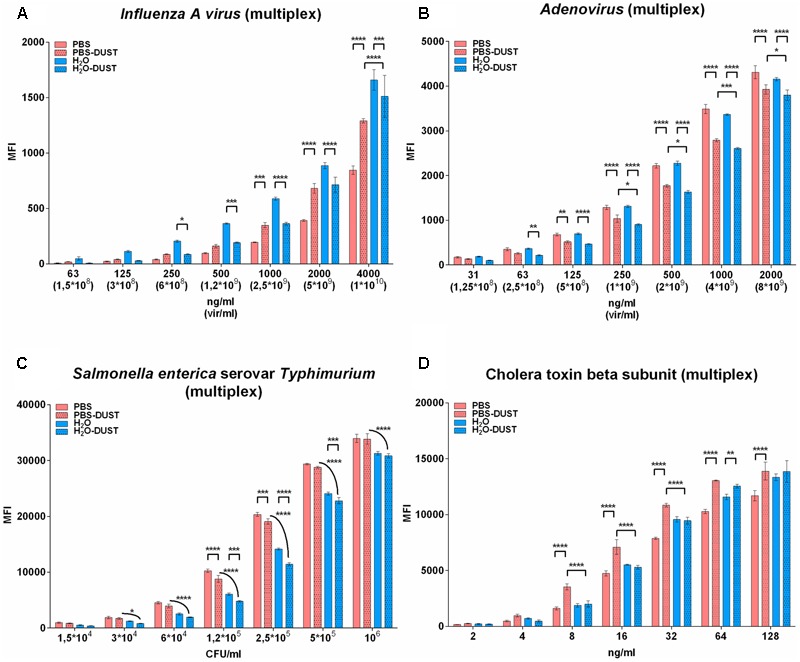
The results of PBA detection in multiplex in different matrices. Result for influenza A virus subtype H5N2 **(A)**, adenovirus type 6 strain Tonsil 99 **(B)**, *S. typhimurium* **(C)** and CTB **(D)** are shown. The data are presented as the mean of three replicates, from which one standard deviation is postponed. Each mean value of the PBA of every matrix was normalized relative to each other by subtracting the corresponding LOD value. Statistical analysis was carried out by the method of multiple comparisons using the Tukey criterion. The only significance between PBS against PBS-DUST, H_2_O against H_2_O-DUST and PBS-DUST against H_2_O-DUST are shown. Other significance bars are hidden for convenience. Statistical significance is: ^∗^*p* < 0.05; ^∗∗^*p* < 0.01; ^∗∗∗^*p* < 0.001; ^∗∗∗∗^*p* < 0.0001. The groups to be compared are indicated by the endings of arcs and staples.

For three of four targets, PBS significantly improved the MFI compared to water. Only for the NP of influenza A the water gave a better MFI for the whole range of concentrations. Dust decreased the MFI in PBS conditions for LPS and AdVH but significantly improved the NP of IAV and CTB toxin in the high concentration range. For low concentrations, dust did not significantly decrease the MFI. For water conditions, the presence of dust negatively affected the MFI (**Figures 2A–C**) or had no effect at all in low ranges of concentrations (**Figure 2D**). Therefore, PBS could be recommended as the buffer of choice for the detection of PBAs in bioaerosol mixtures in the presence of dust.

### Comparison of Mono- vs. Multiplex Detection in the Presence of Dust

**FIGURE 3:**
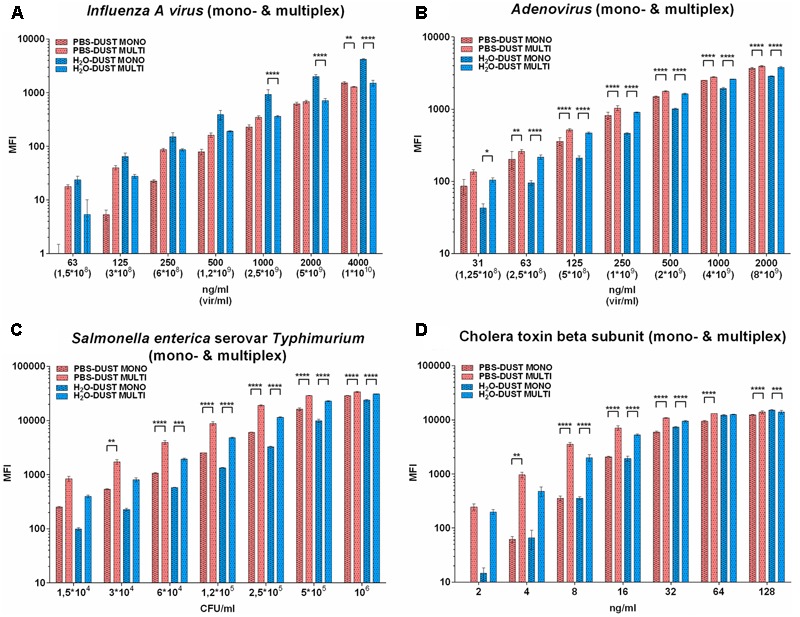
The results of PBA detection in mono- and multiplex regimens in dust-containing matrices. Results for influenza A virus subtype H5N2 **(A)**, adenovirus type 6 strain Tonsil 99 **(B)**, *S. typhimurium* **(C)** and CTB **(D)** are shown. The data are presented as the mean of three replicates, from which one standard deviation is postponed. Each mean value of the PBA of every matrix was normalized relatively each other by subtracting the corresponding LOD value from it. The only significance between PBS-DUST monoplex against PBS-DUST multiplex, H_2_O-DUST monoplex against H_2_O-DUST multiplex are shown. Other significance bars are hidden for convenience. Statistical significance is: ^∗^*p* < 0.05; ^∗∗^*p* < 0.01; ^∗∗∗^*p* < 0.001; ^∗∗∗∗^*p* < 0.0001. The groups to be compared are indicated by the endings of arcs and staples.

To evaluate the effect of dust in monoplex *vs.* multiplex regimens, MFI values in PBS and water in the presence of dust were combined on separate plots. Compared to monoplex conditions, raw MFI LOD was 1.5–3 times higher (**Supplementary Figure S6**) but after subtraction of FT, this trend was no longer seen (**Figure 3**).

Multiplexing of the influenza A virus did not change the MFI in PBS with dust and significantly worsened the values in water with dust at a higher concentration range (**Figure 3A**). In the multiplex conditions for AdVH and STm LPS, the sensitivity limit was similar to the monoplex for all the matrices (**Figures 3B,C**). In the case of CTB detection, PBS-DUST was also the preferred matrix for both sensitivity and MFI levels.

Therefore, multiplexing significantly improved the values for all targets in PBS with the presence of dust compared to monoplex. It is possible to suggest that a non-specific effect of dust in the multiplex mixture is distributed between the four populations of microspheres thus decreasing the effect of dust to individual bead targets.

### Specificity and Precision of the aiRDeTeX 1.0 Test System

To evaluate the analytical properties of aiRDeTeX 1.0, cross-reactivity (**Table 1**) and within-run precision (**Table 2**) were tested for all targets. For cross-reactivity of aiRDeTeX 1.0, the MFI values for all targets were assessed in all individual monoplex assays in triplicate (**Table 1**). Although it would be better to study cross-reactivity over the whole range of concentrations, in present research only highest concentrations of each target had been used, according to some previous studies (Tamborrini et al., 2010; Silbereisen et al., 2015). The value of MFI for every target is significantly higher only in the respective immunoassay. This finding indicates that aiRDeTeX 1.0 specifically detects different targets and does not show cross-reactivity.

**Table 1:** Cross-reactivity of the aiRDeTeX 1.0 system in PBS matrix.

Assays	Targets			
	IAV (4000 ng/ml)	AdV6 (2000 ng/ml)	STm (10^6^ CFU/ml)	CTB (128 ng/ml)
Influenza A virus NP	**934**	33	40	27
Adenovirus type 6 hexon	77	**4614**	75	83
*S. typhimurium* LPS	25	26	**29184**	23
Cholera toxin beta-subunit	54	40	37	**11137**

*The bold values represent target-to-self-monoplex specific signal levels.*

**Table 2:** Within-run precision of aiRDeTeX 1.0 in PBS matrix for the whole range of concentrations.

Influenza A virus NP		Adenovirus type 6 hexon		*S. typhimurium* LPS		Cholera toxin beta-subunit	
Concentration (ng/ml)	CV%	Concentration (ng/ml)	CV%	Concentration (CFU/ml)	CV%	Concentration (ng/ml)	CV%
62.5	2	31	6	1.5^∗^10^4^	10	2	5
125	3	62.5	7	3^∗^10^4^	9	4	10
250	3	125	4	6^∗^10^4^	5	8	7
500	3	250	3	1.2^∗^10^5^	3	16	5
1000	1	500	2	2.5^∗^10^5^	2	32	1
2000	1	1000	3	5^∗^10^5^	0.4	64	2
4000	4	2000	3	10^6^	2	128	4

To evaluate the precision of aiRDeTeX1.0, the coefficient of variation (CV) was calculated for the whole range of concentrations. CV was calculated as the SD divided by the mean of three repetitions in frame of one plate and multiplied to 100%. The within-run precision of aiRDeTeX1.0 was not higher than 10% (**Table 2**).

### Effect of Ultrasound for Multiplex Detection

**FIGURE 4:**
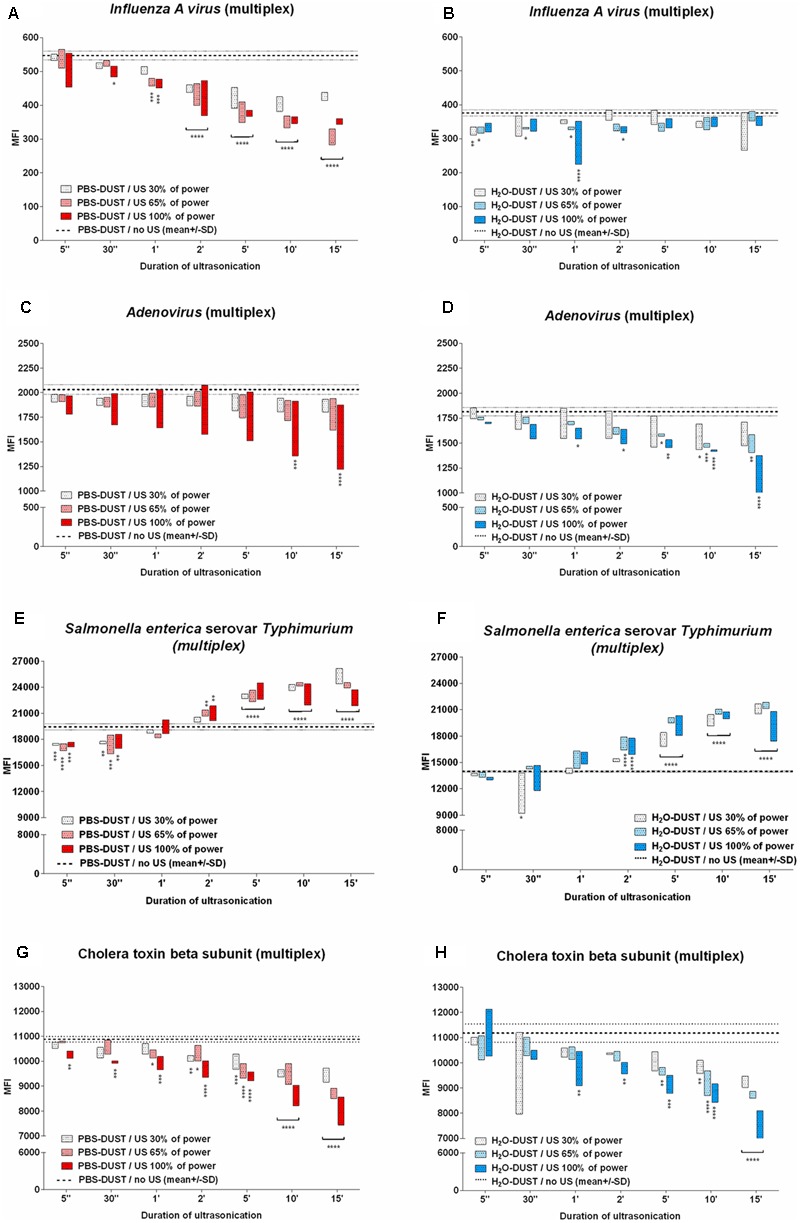
The US effect on PBA detection in dust-containing matrices. Results for influenza A virus subtype H5N2 **(A,B)**, adenovirus type 6 (AdV6) strain Tonsil 99 **(C,D)**, *S. typhimurium* **(E,F)** and CTB **(G,H)** are shown. The effect of ultrasonication for PBS **(A,C,E,G)** and water **(B,D,F,H)** is indicated. The data are presented as floating bars of three replicates (min to max). Statistical analysis between different points after sonication was carried out by the method of multiple comparisons using the Tukey criterion (**Supplementary Figures S7–S10**). The results of comparison values after ultrasonication with values without ultrasonication using the Dunnett test for multiple comparisons with the control group are indicated on the graph by asterisks. Staples unite values with equal importance relative to the control group. Statistical significance is: ^∗^*p* < 0.05; ^∗∗^p < 0.01; ^∗∗∗^*p* < 0.001; ^∗∗∗∗^*p* < 0.0001.

Ultrasound is one of the few sample preparation approaches that could be used before immunological assay in automatic (flow through) bioaerosol surveillance systems as a method for cell lysis and inactivation effect on PBAs (Joyce et al., 2011; Wu et al., 2012). This method seems to be particularly useful for hidden targets (the internal viral NP IAV) or bacterial antigen release (LPS). To assess the effect of ultrasound conditions on multiplex detection, different intensiveness (30, 65, 100%) and times (5″, 30″, 1′, 2′, 5′, 10′, 15′) were tested (**Figure 4**). The results showed that for three out of four targets, ultrasound treatment decreased the MFI (**Figures 4A–D,G,H**). Statistical analysis between different points after sonication was carried out by the method of multiple comparisons using the Tukey criterion (**Supplementary Figures S7–S10**). Only bacterial LPS antigen shows significant MFI improvement upon treatment for more than 2 min (**Figures 4E,F**). Lower US treatment times decreased the MFI both for PBS and water. According to these data, US could improve bacterial immunodetection performance but intensiveness and treatment times should be carefully assessed for all individual targets. For protein (toxin) or viral detection, the use of US is questionable even for internal targets.

## Discussion

Aerosol-transmitted infectious agents cause the greatest concern for the safety of crowded populations in modern cities. Defense strategies rely on effective diagnostics and treatment, but environmental surveillance could provide additional tools for public health and biodefense.

The aim of the present study was to show the possibility of multiplex detection of four different classes of pathogens, including RNA virus (internal NP of influenza A virus), DNA virus (surface hexon of Adenovirus 6), gram-negative bacteria (LPS of *Salmonella* spp.) and toxin (CTB), as model organisms of biologically hazardous components present or potentially spreadable through the air. Therefore, the aiRDeTeX 1.0 model multiplex test system has been developed. It was demonstrated that the raw MFI for aiRDeTeX1.0 was 1.5–3 times higher compared to individual monoplex assays (**Supplementary Figure S6**), but after subtraction of FT, this difference was no longer observed (**Figure 3**).

Individual and multiplex detection of selected antigens was comparable to the best published results for bacteria 10^3^–10^4^ CFU/ml (Kim et al., 2010) and toxin 2 ng/ml (Garber et al., 2010) but much less for viral targets corresponding to 10^8^ viral particles/ml both for internal NP and surface AdVH (**Figures 1, 2**).

According to previous studies, less than 1.6^∗^10^4^ influenza particles might be enough to cause infection (Nikitin et al., 2014). Similar number of virions is present in 1 m^3^ of air during influenza season (Yang et al., 2011). If particles are collected from 20 m^3^ of air into 4 ml of buffer, then sensitivity better that 5^∗^10^4^ vir/ml needs to be achieved to reliably detect influenza threat. According to these calculations, achieved sensitivity of 10^8^ vir/ml is clearly insufficient. However, if we look from biosafety perspective, the PBA concentrations might be greater than 10^4^ vir/ml in case of a bioterrorist attack, and even lower-sensitivity test-systems can still be useful under such conditions.

Prolonged sample collection time can be used to improve virus detection. Detection of internal nucleocapsid targets could also be improved by buffer condition optimization. It is further possible that better sensitivity might be achieved by using better Abs. On the other hand, the sensitivity of viruses detection is comparable to that of toxins detection if it is measured in mass values rather than virions. According to several papers (Garber et al., 2010; Simonova et al., 2012) describing toxin detection by xMAP technology, the sensitivity is typically around few nanograms/ml. This is still not enough for viruses because 1 ng/ml of the virus roughly corresponds to 10^6^ vir/ml. Some cytokine MIA test-systems have working range of 7.8–4000 ng/L (Skogstrand et al., 2005), which corresponds to approximately 10^4^ vir/ml of influenza. However, cytokine detection protocols require overnight incubation, which is not acceptable for prompt biosurveillance analyses.

Thus, sensitivity higher than 10^4^ vir/ml relevant to nasal influenza infection is hardly achievable with xMAP technique and requires very good antibodies, extensive protocol optimization, prolonged sample collection and incubation times. To detect such a low viral load, Luminex xTAG technique based on nucleic acid amplification could be used. Last year Luminex released xTAG^®^ RESPIRATORY VIRAL PANEL including influenza A and B. Such systems could be very useful for environmental biosurveillance but they do not allow toxin detection and its applicability for pathogen detection directly in environmental samples is not yet studied. Nucleic acid extraction step could be obligatory to remove polymerase inhibitors.

Nevertheless, the described in the present paper model multiplex panel was suitable to study the effect of matrix (PBS vs. water), the presence of dust and ultrasound treatment for detection of targets. Non-specific interactions of aiRDeTeX 1.0 are around FT (**Table 1**), and CV is less than 10% for the whole range of concentrations (**Table 2**).

The type of solution and presence of dust differently affected the MFI of diverse targets at a specific concentration range. Therefore, such variation of MFI leads to a decrease in the fluorescence threshold for IAV rate (**Figure 2A**; multiplex in PBS) or toxin (**Figure 1D**; monoplex in water). Nevertheless, trade-offs led us to conclude that PBS is generally better than water in three out of four cases in monoplex (**Figure 1**) and in the presence of dust (**Figure 3**). It is important that in the low range of concentrations in the presence of dust, PBS showed similar or better results compared to water. It is possible that the non-specific effect of dust in multiplex mixture is distributed between four populations of microspheres, thus decreasing the effect of dust to individual bead targets.

Ultrasound treatment is thought to increase the concentration of antigen containing particles for bacteria and promotes internal antigen release. The present study shows that the use of US treatment is not unequivocally. Therefore, in three out of four targets, US was either neutral or significantly decreased the MFI (**Figure 4**). Bacterial LPS is the only antigen where the MFI increases correlates with the increases in US intensity and of treatment times. Thus, ultrasound treatment was useful for the detection of Salmonella LPS (**Figure 4**). Unfortunately, in frame of the present study, the sensitivity change due to US treatment was not studied. A sensitivity of detection of 10^3^–10^4^ CFU/ml without US treatment was obtained. Hypothetically, in case of US treatment use, a shift to PCR attainable 10^2^–10^3^ CFU/ml sensitivity could be achieved.

The chemical, physical and biological properties of dust as part of aerosol could be significantly different (Macher et al., 2012). Thus, diverse samples of aerosol dust collected in random places might differently affect MIA. The same trend was reported for food matrices (Kim et al., 2010). In line with the present study, only one type of aerosol dust was used for all dust experiments for consistency of the results. To evaluate the effect of particular dust properties on MIA, it is necessary to study more samples with detailed dust characterization. AFM and electronic microscopy, zeta potential, elementary and molecular content should be studied to differentiate and categorize dust samples and clarify the major factors in MIA performance.

## Author Contributions

Conceived and designed the experiments: DK, VG, and AT. Performed the experiments: DK. Analyzed and interpreted the data: DK and VG. Wrote the paper: DK and VG. AG, DK, VG, AT critically reviewed and revised the manuscript. All the authors read and approved the final manuscript.

## Conflict of Interest Statement

The authors declare that the research was conducted in the absence of any commercial or financial relationships that could be construed as a potential conflict of interest.

## Supplementary Material

FIGURE S1 Examples of optimization: some parameters of the adenovirus monoplex. Results of some parameters optimization of AdV6-detection test-system are shown. Factor of different concentrations of coupling ABs, conjugated with beads was studied using anti-mouse mABs in coupling confirmation procedure (Luminex, 2016) **(A)**, and in full length assay **(B)**; different incubation times, shaking intensity and temperature regimens were optimized **(C)**.

FIGURE S2 Examples of optimization: some parameters of the salmonella monoplex. Results of some parameters optimization of STm-detection test-system are shown. Factor of different concentrations of coupling ABs, conjugated with beads was studied using anti-mouse mABs in coupling confirmation procedure (Luminex, 2016) **(A)**, and in full length assay **(B)**; different incubation times, shaking intensity and temperature regimens were optimized **(C)**.

FIGURE S3 Optimization of blocking conditions. Results of coupled beads blocking optimization of AdV6 **(A)** and STm **(B)** – detection test-system are shown.

FIGURE S4 Results of different lysing component usage for NP extraction from IAV virion.

FIGURE S5 The results of STm detection in monoplex for all four matrices (buffer condition). Results of *S. typhimurium* detection in monoplex are shown. The data are presented as the mean of three replicates, from which one standard deviation is postponed. Each mean value of the STm of every matrix was normalized relative to each other by subtracting the corresponding LOD value. Statistical analysis was carried out by the method of multiple comparisons using the Tukey criterion. The only significance between PBS against PBS-DUST, H_2_O against H_2_O-DUST and PBS-DUST against H_2_O-DUST is shown. Other significance bars are hidden for convenience. Statistical significance is: ^∗^*p* < 0.05; ^∗∗^*p* < 0.01; ^∗∗∗^*p* < 0.001; ^∗∗∗∗^*p* < 0.0001. The groups being compared are indicated by the endings of arcs and staples.

FIGURE S6 The results of PBA detection in mono- and multiplex in dust-containing matrices. Results of influenza A virus subtype H5N2 **(A)**, adenovirus type 6 (AdV6) strain Tonsil 99 **(B)**, *S. typhimurium* **(C)** and CTB **(D)** are shown. The data are presented as the mean of three replicates, from which one standard deviation is postponed. Mean value of the PBA of every matrix wasn’t normalized compare to **Figure 3**. The only significance between PBS-DUST monoplex against PBS-DUST multiplex, H_2_O-DUST monoplex against H_2_O-DUST multiplex is shown. Other significance bars are hidden for convenience. Statistical significance is: ^∗^*p* < 0.05; ^∗∗^*p* < 0.01; ^∗∗∗^*p* < 0.001; ^∗∗∗∗^*p* < 0.0001. The groups to be compared are indicated by the endings of arcs and staples.

FIGURE S7 Statistical analysis between different points of Influenza A virus nucleoprotein detection after sonication. Results of multiple comparisons of IAV detection every point with every point using the Tukey criterion are shown. On **(A,B)** plots results after different duration and power of ultrasonication in PBS-DUST matrix are shown. Same condition points, but in H_2_0-DUST are plotted on **(C,D)** plots. Statistical significance is: ^∗^*p* < 0.05; ^∗∗^*p* < 0.01; ^∗∗∗^*p* < 0.001; ^∗∗∗∗^*p* < 0.0001.

FIGURE S8 Statistical analysis between different points of Adenovirus 6 type hexon detection after sonication. Results of multiple comparisons of AdV6 detection every point with every point using the Tukey criterion are shown. On **(A,B)** plots results after different duration and power of ultrasonication in PBS-DUST matrix are shown. Same condition points, but in H_2_0-DUST are plotted on **(C,D)** plots. Statistical significance is: ^∗^*p* < 0.05; ^∗∗^*p* < 0.01; ^∗∗∗^*p* < 0.001; ^∗∗∗∗^*p* < 0.0001.

FIGURE S9 Statistical analysis between different points of *S. typhimurium* LPS detection after sonication. Results of multiple comparisons of STm detection every point with every point using the Tukey criterion are shown. On **(A,B)** plots results after different duration and power of ultrasonication in PBS-DUST matrix are shown. Same condition points, but in H_2_0-DUST are plotted on **(C,D)** plots. Statistical significance is: ^∗^*p* < 0.05; ^∗∗^*p* < 0.01; ^∗∗∗^*p* < 0.001; ^∗∗∗∗^*p* < 0.0001.

FIGURE S10 Statistical analysis between different points of cholera toxin beta subunit detection after sonication. Results of multiple comparisons of CTB detection every point with every point using the Tukey criterion are shown. On **(A,B)** plots results after different duration and power of ultrasonication in PBS-DUST matrix are shown. Same condition points, but in H_2_0-DUST are plotted on **(C,D)** plots. Statistical significance is: ^∗^*p* < 0.05; ^∗∗^*p* < 0.01; ^∗∗∗^*p* < 0.001; ^∗∗∗∗^*p* < 0.0001.

## References

[B1] Ayouba A. Touré A. Butel C. Keita A. K. Binetruy F. Sow M. S. (2017). Development of a sensitive and specific serological assay based on luminex technology for detection of antibodies to *Zaire ebolavirus*. *J. Clin. Microbiol.* 55 165–176. 10.1128/JCM.01979-16 27795350 PMC5228227

[B2] Barras V. Greub G. (2014). History of biological warfare and bioterrorism. *Clin. Microbiol. Infect.* 20 497–502. 10.1111/1469-0691.12706 24894605

[B3] Centers for Disease Control and Prevention (2017). *Bioterrorism Agents/Diseases*. Available at: https://emergency.cdc.gov/agent/agentlist.asp

[B4] Czeh A. Mandy F. Feher-Toth S. Torok L. Mike Z. Koszegi B. (2012). A flow cytometry based competitive fluorescent microsphere immunoassay (CFIA) system for detecting up to six mycotoxins. *J. Immunol. Methods* 384 71–80. 10.1016/j.jim.2012.07.010 22841575

[B5] Dzenitis J. M. Makarewicz A. J. (2010). “The autonomous pathogen detection system,” in *The Microflow Cytometer* eds Ligler F. S. Kim J. S. (Singapore: Pan Stanford Publishing) 263–286.

[B6] Fulwyler M. J. (1976). Optical Chamber with Spherical Reflective Portion and Apparatus Employing Same. UK Patent No 1561042.

[B7] Garber E. A. Venkateswaran K. V. O’Brien T. W. (2010). Simultaneous multiplex detection and confirmation of the proteinaceous toxins abrin, ricin, botulinum toxins, and *Staphylococcus* enterotoxins a, b, and c in food. *J. Agric. Food Chem.* 58 6600–6607. 10.1021/jf100789n 20455521

[B8] Guo Y. R. Tian J. Liang C. Z. Zhu G. N. Gui W. J. (2013). Multiplex bead-array competitive immunoassay for simultaneous detection of three pesticides in vegetables. *Microchim. Acta* 180 387–395. 10.1007/s00604-013-0944-4

[B9] Hoare R. Thompson K. D. Herath T. Collet B. Bron J. E. Adams A. (2016). Development, characterisation and application of monoclonal antibodies for the detection and quantification of infectious salmon anaemia virus in plasma samples using luminex bead array technology. *PLOS ONE* 11:e0159155. 10.1371/journal.pone.0159155 27434377 PMC4951118

[B10] Joyce E. Al-Hashimi A. Mason T. J. (2011). Assessing the effect of different ultrasonic frequencies on bacterial viability using flow cytometry. *J. Appl. Microbiol.* 110 862–870. 10.1111/j.1365-2672.2011.04923.x 21324052

[B11] Kerkhof K. Canier L. Kim S. Heng S. Sochantha T. Sovannaroth S. (2015). Implementation and application of a multiplex assay to detect malaria-specific antibodies: a promising tool for assessing malaria transmission in Southeast Asian pre-elimination areas. *Malar. J.* 14:338. 10.1186/s12936-015-0868-z 26337785 PMC4558921

[B12] Kim J. S. Taitt C. R. Ligler F. S. Anderson G. P. (2010). Multiplexed magnetic microsphere immunoassays for detection of pathogens in foods. *Sens. Instrum. Food Qual. Saf.* 4 73–81. 10.1007/s11694-010-9097-x 20953301 PMC2953821

[B13] Leirs K. Tewari Kumar P. Decrop D. Pérez-Ruiz E. Leblebici P. van Kelst B. (2016). Bioassay development for ultrasensitive detection of influenza a nucleoprotein using digital ELISA. *Anal. Chem.* 88 8450–8458. 10.1021/acs.analchem.6b00502 27487722

[B14] Luminex (2016). *The xMAP^®^ Cookbook* 3rd Edn. Available at: http://info.luminexcorp.com/en-us/download-the-xmap-cookbook [accessed May 5, 2017].

[B15] Macher J. M. Douwes J. Prezant B. Reponen T. (2012). “Bioaerosols,” in: *Aerosols Handbook Measurement, Dosimetry, and Health Effects* 2nd Edn eds Ruzer L. S. Harley N. H. (Boca Raton, FL: CRC Press) 285–345. 10.1201/b12668-13

[B16] Makarov V. V. Khromov A. V. Guschin V. A. Tkachuk A. P. (2017). Emergence of new infections in the 21st century and identification of pathogens using next generation sequencing. *Bull. RSMU* 1 5–23. 10.24075/brsmu.2017-01-01

[B17] McHugh T. M. (1994). Flow microsphere immunoassay for the quantitative and simultaneous detection of multiple soluble analytes. *Methods Cell Biol.* 42 575–595. 10.1016/S0091-679X(08)61096-1 7877510

[B18] Nikitin N. Petrova E. Trifonova E. Karpova O. (2014). Influenza virus aerosols in the air and their infectiousness. *Adv. Virol.* 2014:859090. 10.1155/2014/859090 25197278 PMC4147198

[B19] Reslova N. Michna V. Kasny M. Mikel P. Kralik P. (2017). xMAP technology: applications in detection of pathogens. *Front. Microbiol.* 8:55 10.3389/fmicb.2017.00055 PMC5263158 28179899

[B20] Silbereisen A. Tamborrini M. Wittwer M. Schürch N. Pluschke G. (2015). Development of a bead-based luminex assay using lipopolysaccharide specific monoclonal antibodies to detect biological threats from *Brucella* species. *BMC Microbiol.* 15:198. 10.1186/s12866-015-0534-531 26438077 PMC4595103

[B21] Simonova M. A. Valyakina T. I. Petrova E. E. Komaleva R. L. Shoshina N. S. Samokhvalova L. V. (2012). Development of xMAP assay for detection of six protein toxins. *Anal. Chem.* 84 6326–6330. 10.1021/ac301525q 22794090

[B22] Skogstrand K. Thorsen P. Nørgaard-Pedersen B. Schendel D. E. Sørensen L. C. Hougaard D. M. (2005). Simultaneous measurement of 25 inflammatory markers and neurotrophins in neonatal dried blood spots by immunoassay with xMAP technology. *Clin. Chem.* 51 1854–1866. 10.1373/clinchem.2005.052241 16081507

[B23] Smirnov Y. A. Lipatov A. S. Van Beek R. Gitelman A. K. Osterhaus A. D. Claas E. C. (2000). Characterization of adaptation of an avian influenza A (H5N2) virus to a mammalian host. *Acta Virol.* 44 1–8. 10989685

[B24] Tamborrini M. Holzer M. Seeberger P. H. Schürch N. Pluschke G. (2010). Anthrax spore detection by a luminex assay based on monoclonal antibodies that recognize anthrose-containing oligosaccharides. *Clin. Vaccine Immunol.* 17 1446-1451. 10.1128/CVI.00205-210. 20660139 PMC2944465

[B25] Wu X. Joyce E. M. Mason T. J. (2012). Evaluation of the mechanisms of the effect of ultrasound on *Microcystis aeruginosa* at different ultrasonic frequencies. *Water Res.* 46 2851–2858. 10.1016/j.watres.2012.02.019 22440593

[B26] Wynwood S. J. Burns M. A. Graham G. C. Weier S. L. McKay D. B. Craig S. B. (2015). Validation of a microsphere immunoassay for serological leptospirosis diagnosis in human serum by comparison to the current gold standard. *PLOS Negl. Trop. Dis.* 9:e0003636. 10.1371/journal.pntd.0003636 25807009 PMC4373873

[B27] Yang W. Elankumaran S. Marr L. (2011). Concentrations and size distributions of airborne influenza a viruses measured indoors at a health centre, a day-care centre and on aeroplanes. *J. R. Soc. Interface* 8 1176–1184. 10.1098/rsif.2010.0686 21300628 PMC3119883

