# Supplementary figures and images for: Impact of Aerosol Dust on xMAP Multiplex Detection of Different Class Pathogens
Source: Front Microbiol. 2017 Nov 29;8:2341. doi: 10.3389/fmicb.2017.02341 (PMC5712594; doi:10.3389/fmicb.2017.02341)

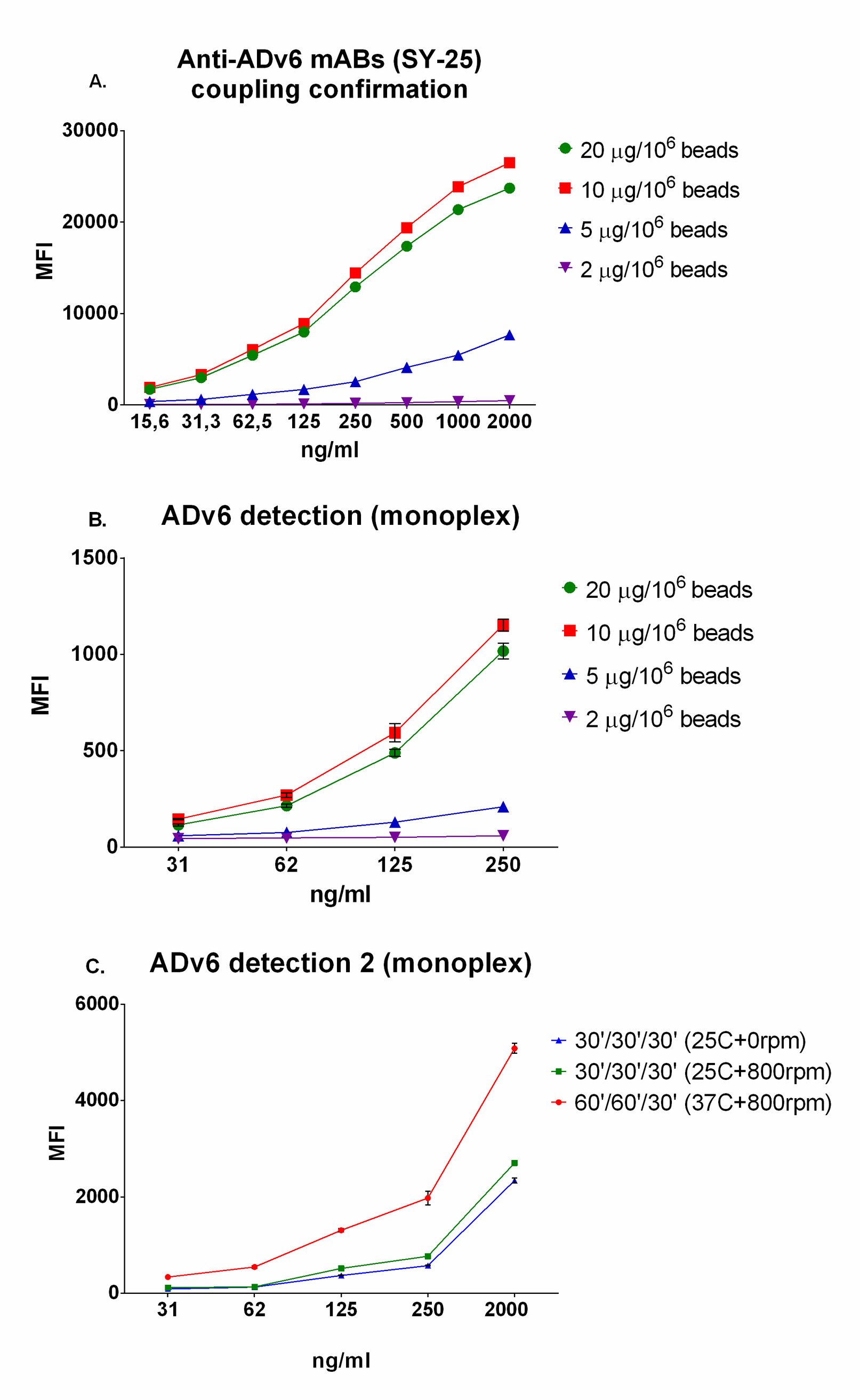

Supplement: FIGURE S1 — Examples of optimization: some parameters of the adenovirus monoplex. Results of some parameters optimization of AdV6-detection test-system are shown. Factor of different concentrations of coupling ABs, conjugated with beads was studied using anti-mouse mABs in coupling confirmation procedure (Luminex, 2016) (A), and in full length assay (B); different incubation times, shaking intensity and temperature regimens were optimized (C). [file Image_1.TIF]

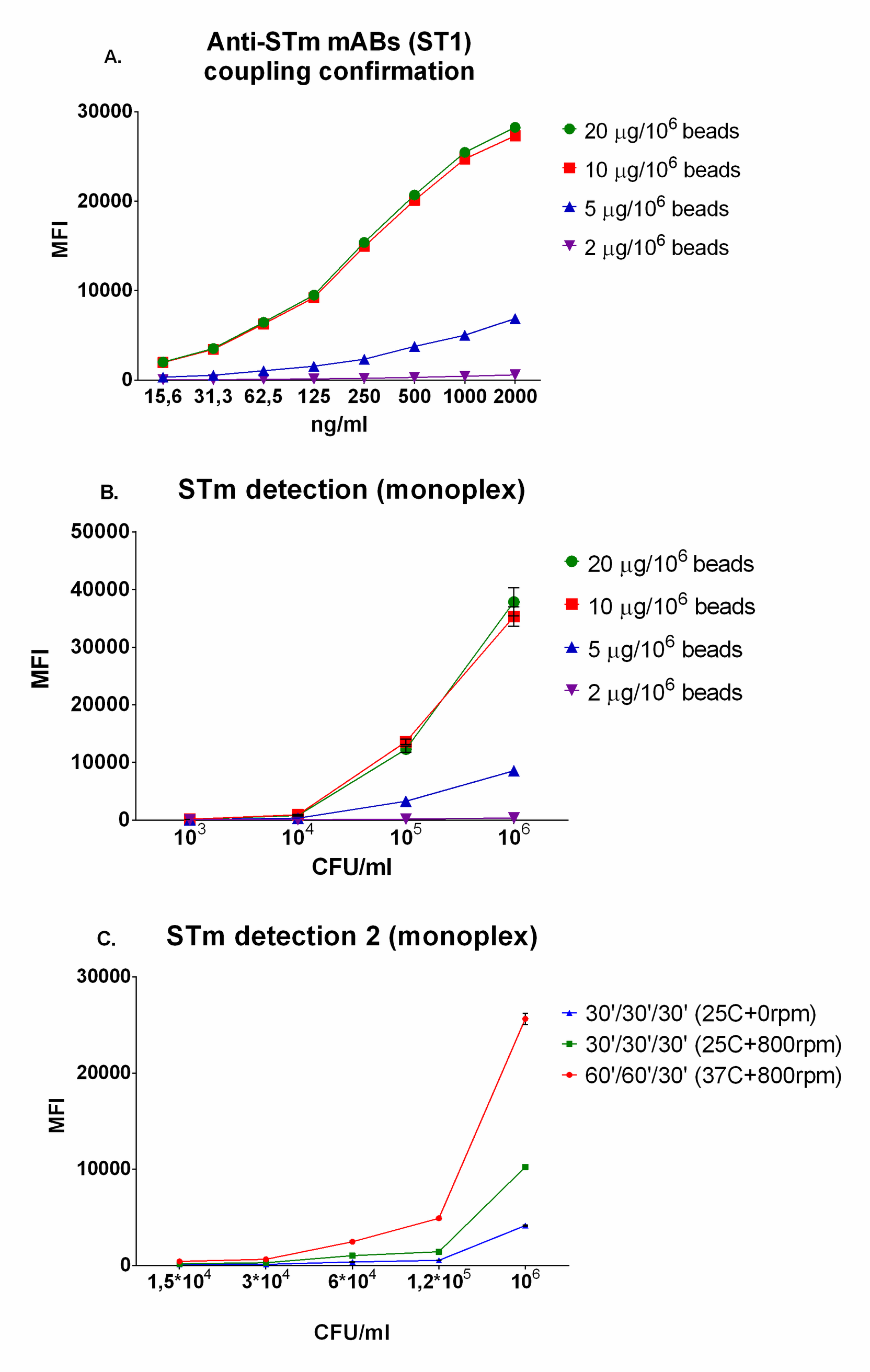

Supplement: FIGURE S2 — Examples of optimization: some parameters of the salmonella monoplex. Results of some parameters optimization of STm-detection test-system are shown. Factor of different concentrations of coupling ABs, conjugated with beads was studied using anti-mouse mABs in coupling confirmation procedure (Luminex, 2016) (A), and in full length assay (B); different incubation times, shaking intensity and temperature regimens were optimized (C). [file Image_2.TIF]

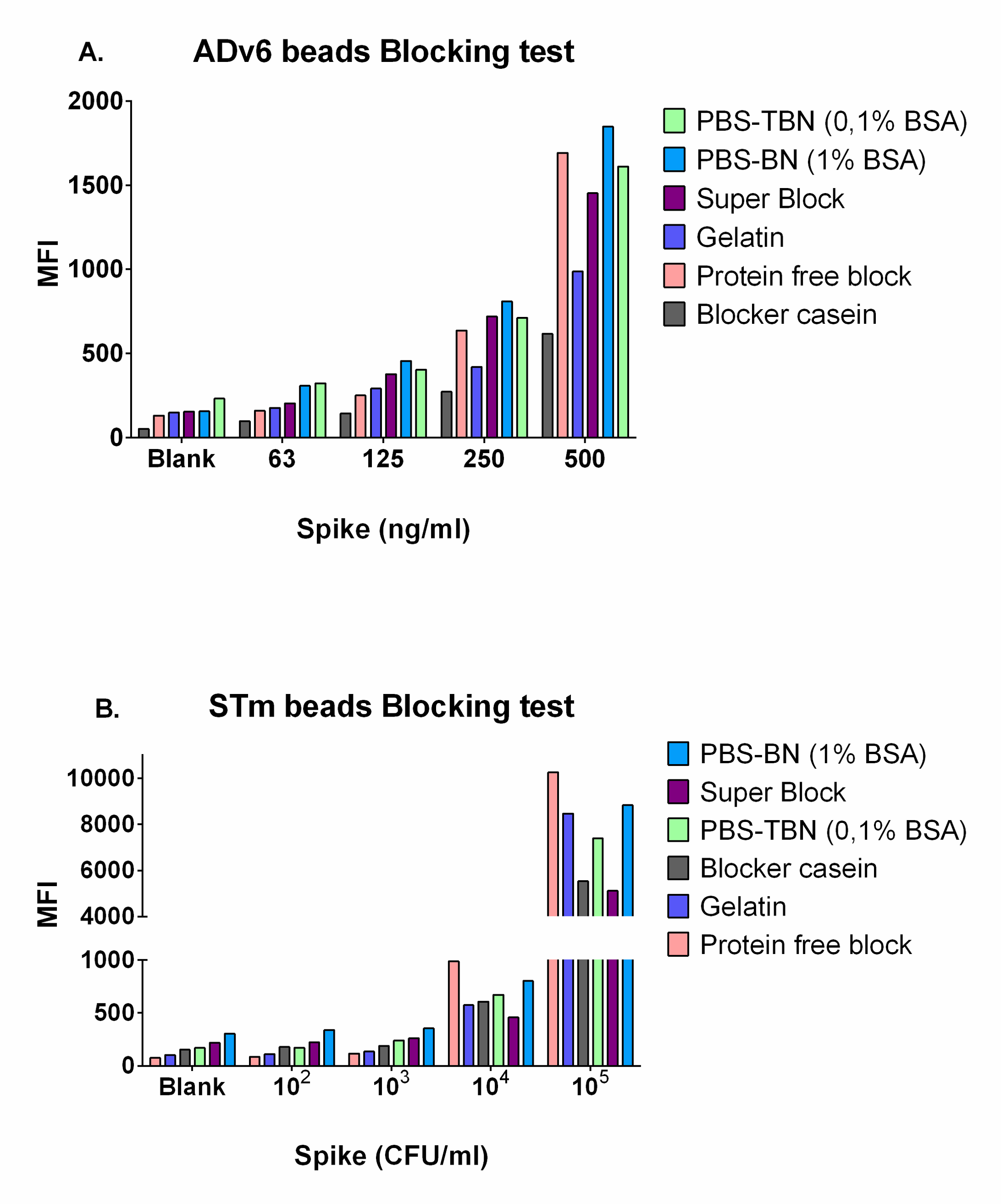

Supplement: FIGURE S3 — Optimization of blocking conditions. Results of coupled beads blocking optimization of AdV6 (A) and STm (B) – detection test-system are shown. [file Image_3.TIF]

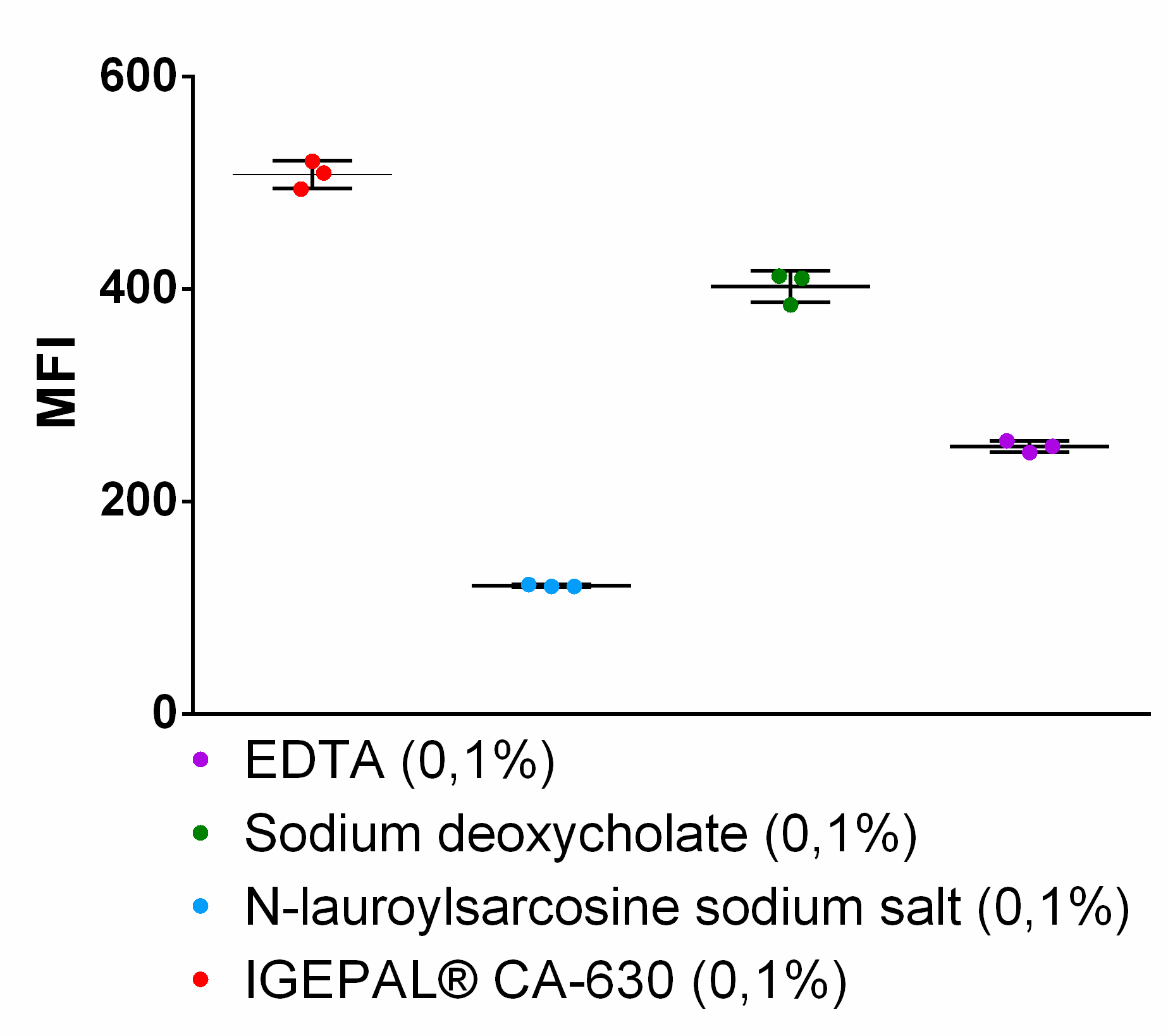

Supplement: FIGURE S4 — Results of different lysing component usage for NP extraction from IAV virion. [file Image_4.TIF]

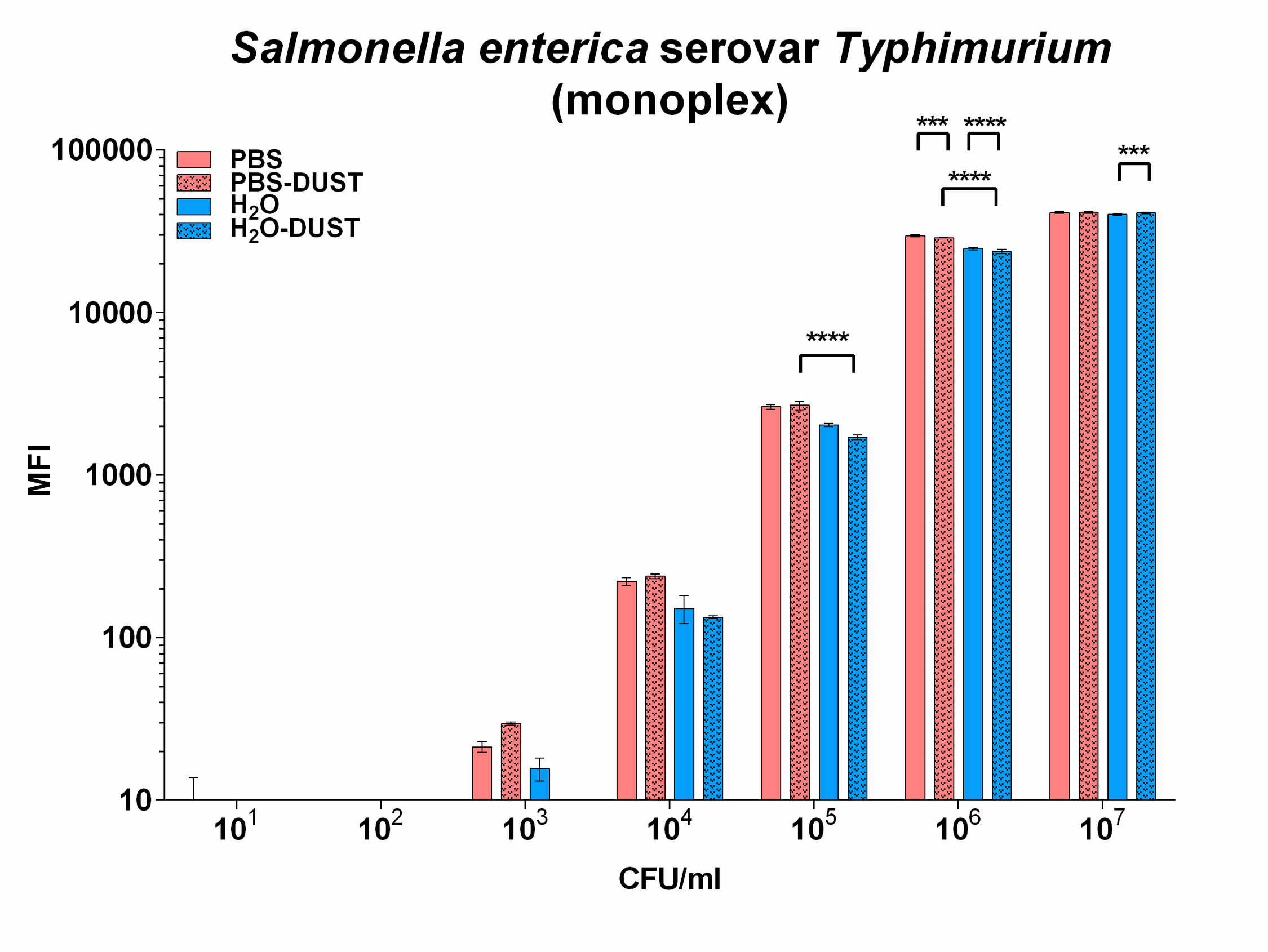

Supplement: FIGURE S5 — The results of STm detection in monoplex for all four matrices (buffer condition). Results of S. typhimurium detection in monoplex are shown. The data are presented as the mean of three replicates, from which one standard deviation is postponed. Each mean value of the STm of every matrix was normalized relative to each other by subtracting the corresponding LOD value. Statistical analysis was carried out by the method of multiple comparisons using the Tukey criterion. The only significance between PBS against PBS-DUST, H2O against H2O-DUST and PBS-DUST against H2O-DUST is shown. Other significance bars are hidden for convenience. Statistical significance is: ∗p < 0.05; ∗∗p < 0.01; ∗∗∗p < 0.001; ∗∗∗∗p < 0.0001. The groups being compared are indicated by the endings of arcs and staples. [file Image_5.TIF]

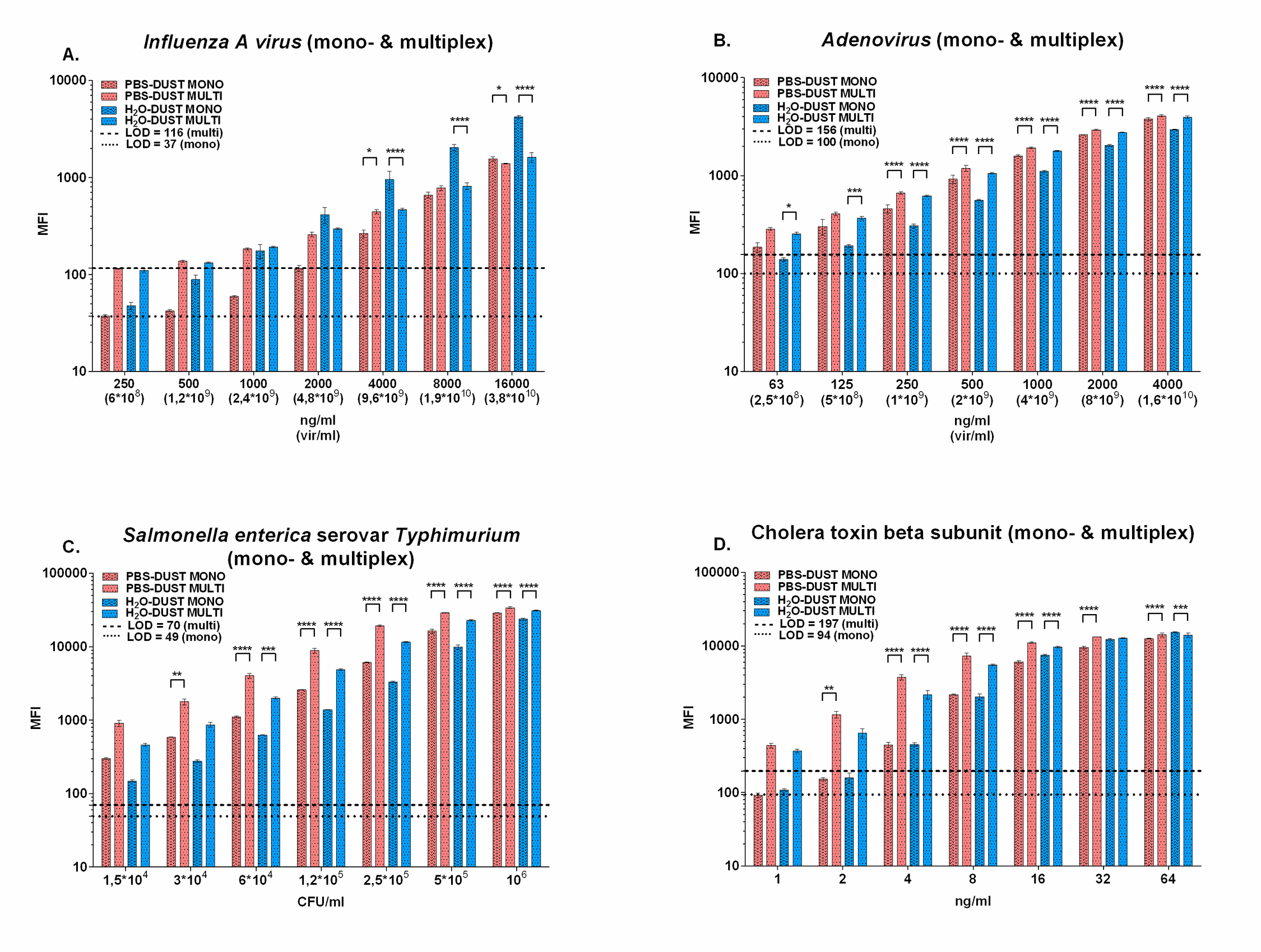

Supplement: FIGURE S6 — The results of PBA detection in mono- and multiplex in dust-containing matrices. Results of influenza A virus subtype H5N2 (A), adenovirus type 6 (AdV6) strain Tonsil 99 (B), S. typhimurium (C) and CTB (D) are shown. The data are presented as the mean of three replicates, from which one standard deviation is postponed. Mean value of the PBA of every matrix wasn’t normalized compare to Figure 3. The only significance between PBS-DUST monoplex against PBS-DUST multiplex, H2O-DUST monoplex against H2O-DUST multiplex is shown. Other significance bars are hidden for convenience. Statistical significance is: ∗p < 0.05; ∗∗p < 0.01; ∗∗∗p < 0.001; ∗∗∗∗p < 0.0001. The groups to be compared are indicated by the endings of arcs and staples. [file Image_6.TIF]

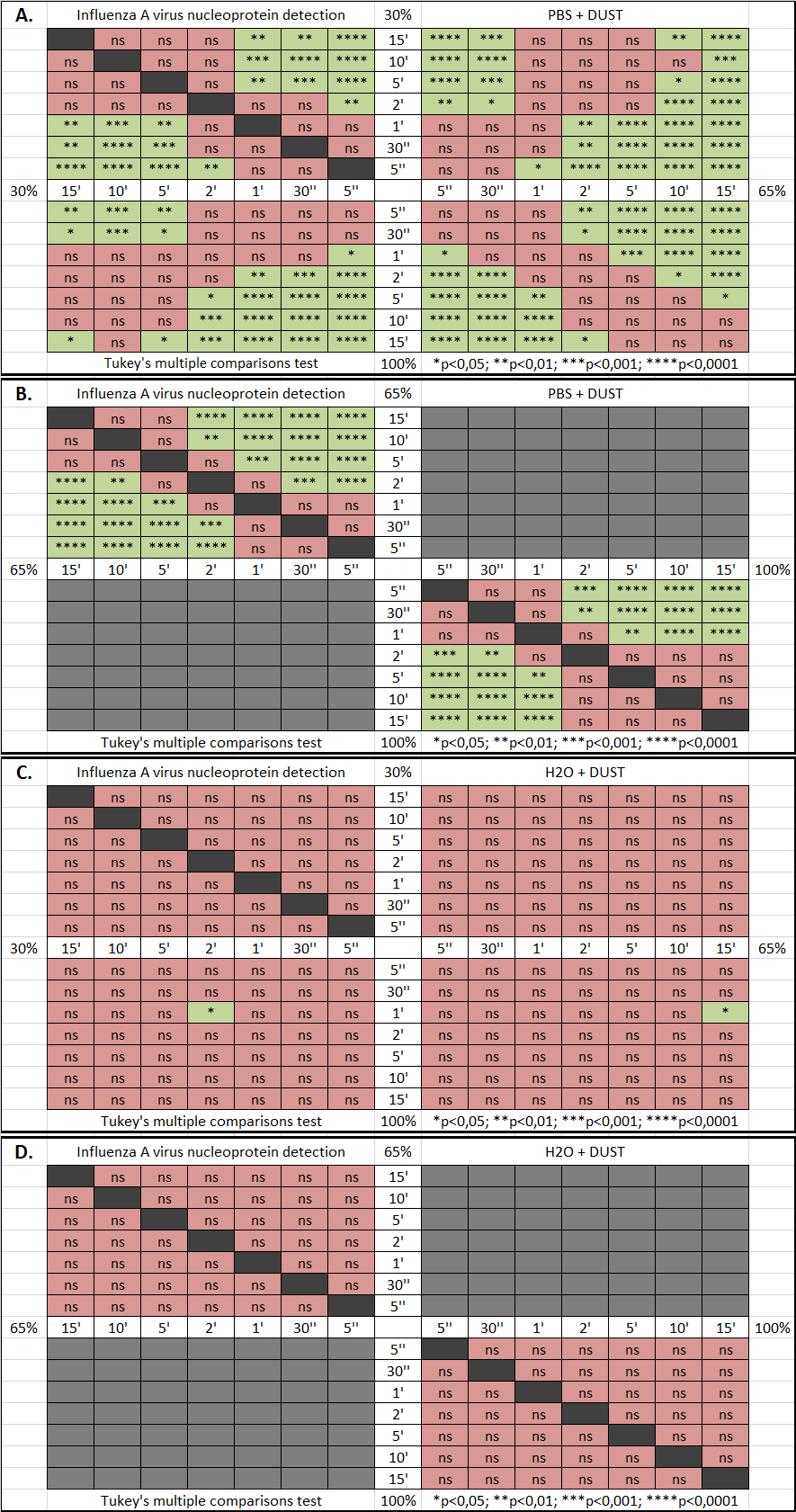

Supplement: FIGURE S7 — Statistical analysis between different points of Influenza A virus nucleoprotein detection after sonication. Results of multiple comparisons of IAV detection every point with every point using the Tukey criterion are shown. On (A,B) plots results after different duration and power of ultrasonication in PBS-DUST matrix are shown. Same condition points, but in H20-DUST are plotted on (C,D) plots. Statistical significance is: ∗p < 0.05; ∗∗p < 0.01; ∗∗∗p < 0.001; ∗∗∗∗p < 0.0001. [file Image_7.TIF]

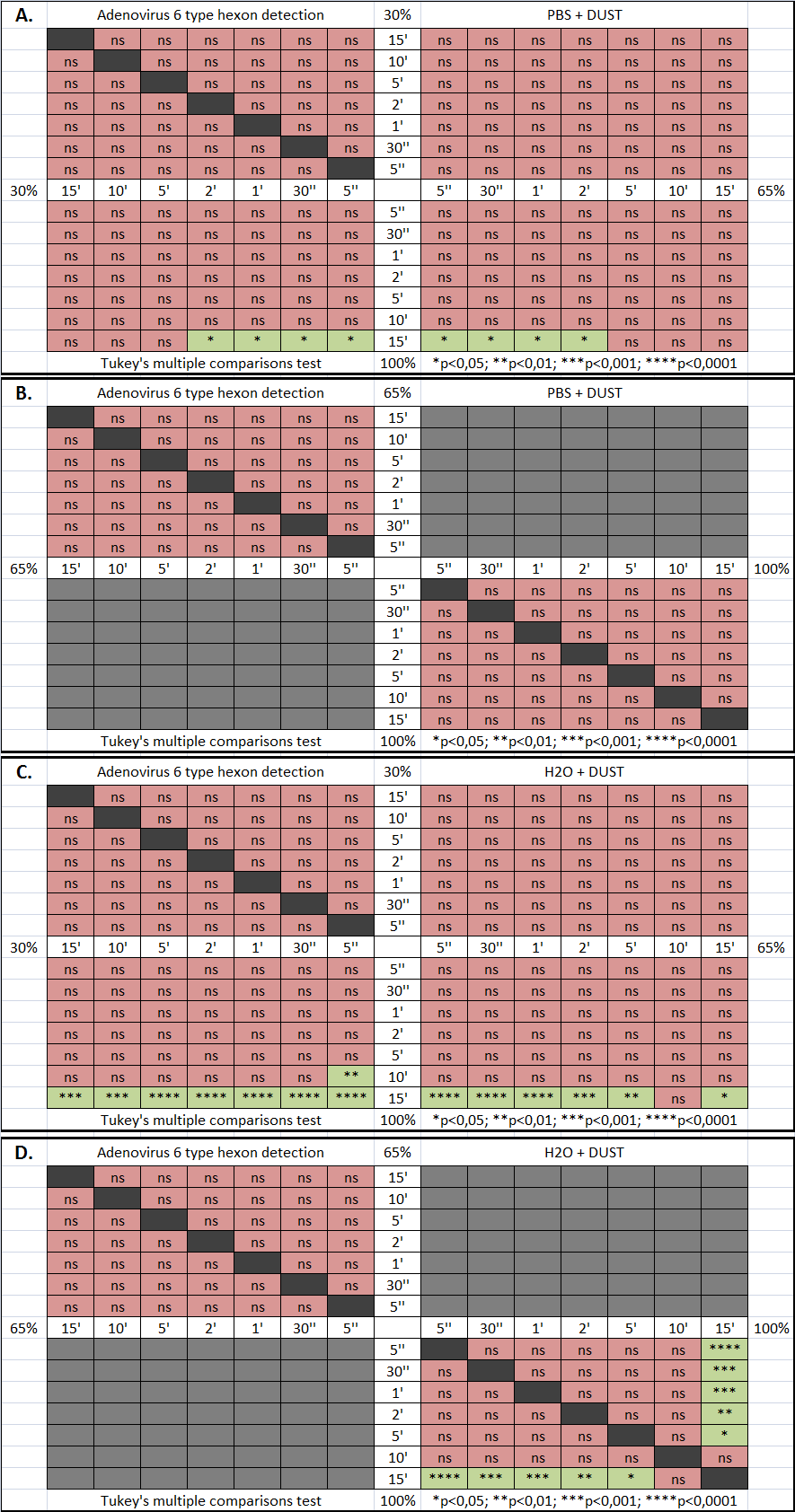

Supplement: FIGURE S8 — Statistical analysis between different points of Adenovirus 6 type hexon detection after sonication. Results of multiple comparisons of AdV6 detection every point with every point using the Tukey criterion are shown. On (A,B) plots results after different duration and power of ultrasonication in PBS-DUST matrix are shown. Same condition points, but in H20-DUST are plotted on (C,D) plots. Statistical significance is: ∗p < 0.05; ∗∗p < 0.01; ∗∗∗p < 0.001; ∗∗∗∗p < 0.0001. [file Image_8.TIF]

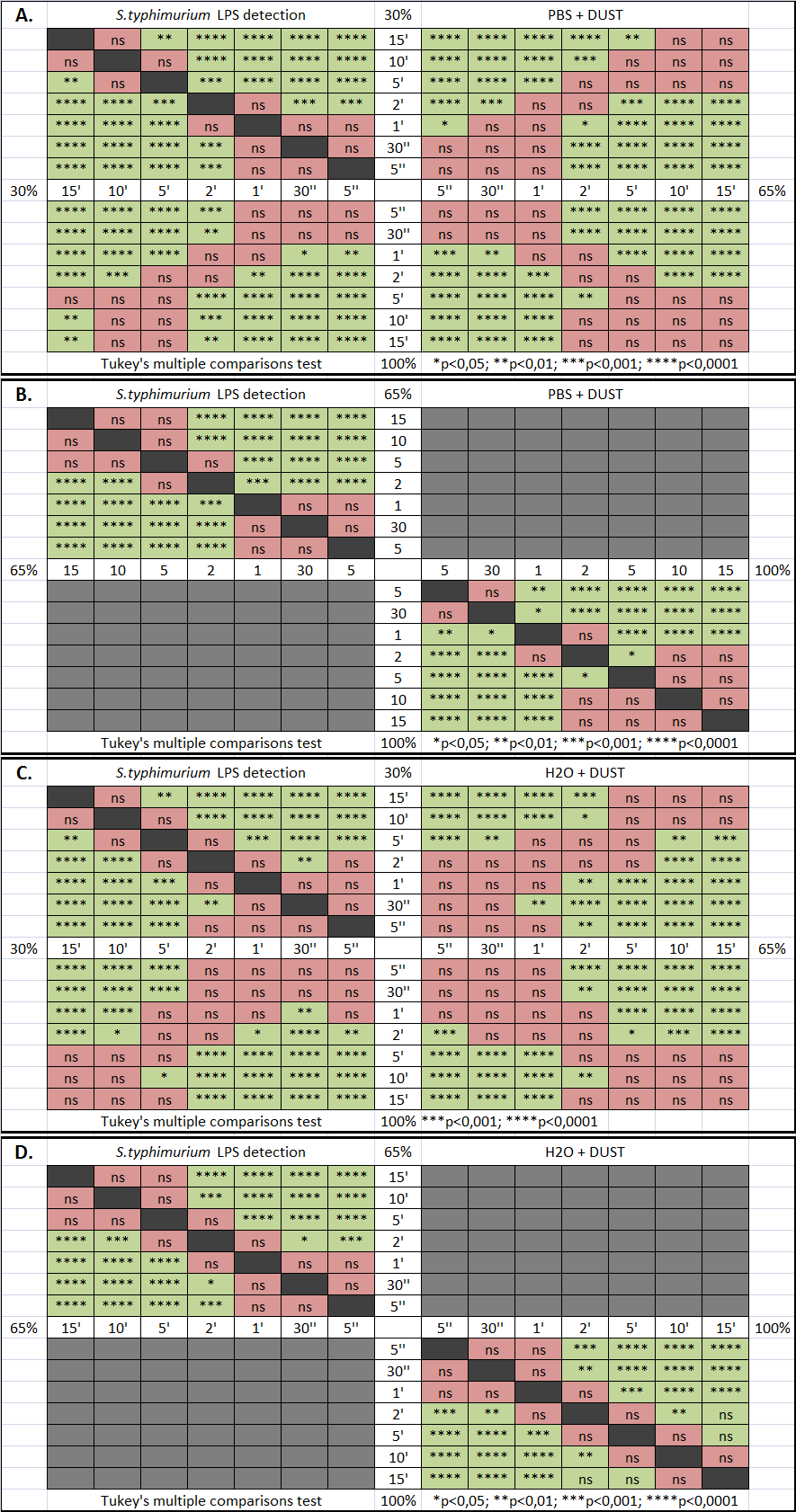

Supplement: FIGURE S9 — Statistical analysis between different points of S. typhimurium LPS detection after sonication. Results of multiple comparisons of STm detection every point with every point using the Tukey criterion are shown. On (A,B) plots results after different duration and power of ultrasonication in PBS-DUST matrix are shown. Same condition points, but in H20-DUST are plotted on (C,D) plots. Statistical significance is: ∗p < 0.05; ∗∗p < 0.01; ∗∗∗p < 0.001; ∗∗∗∗p < 0.0001. [file Image_9.TIF]

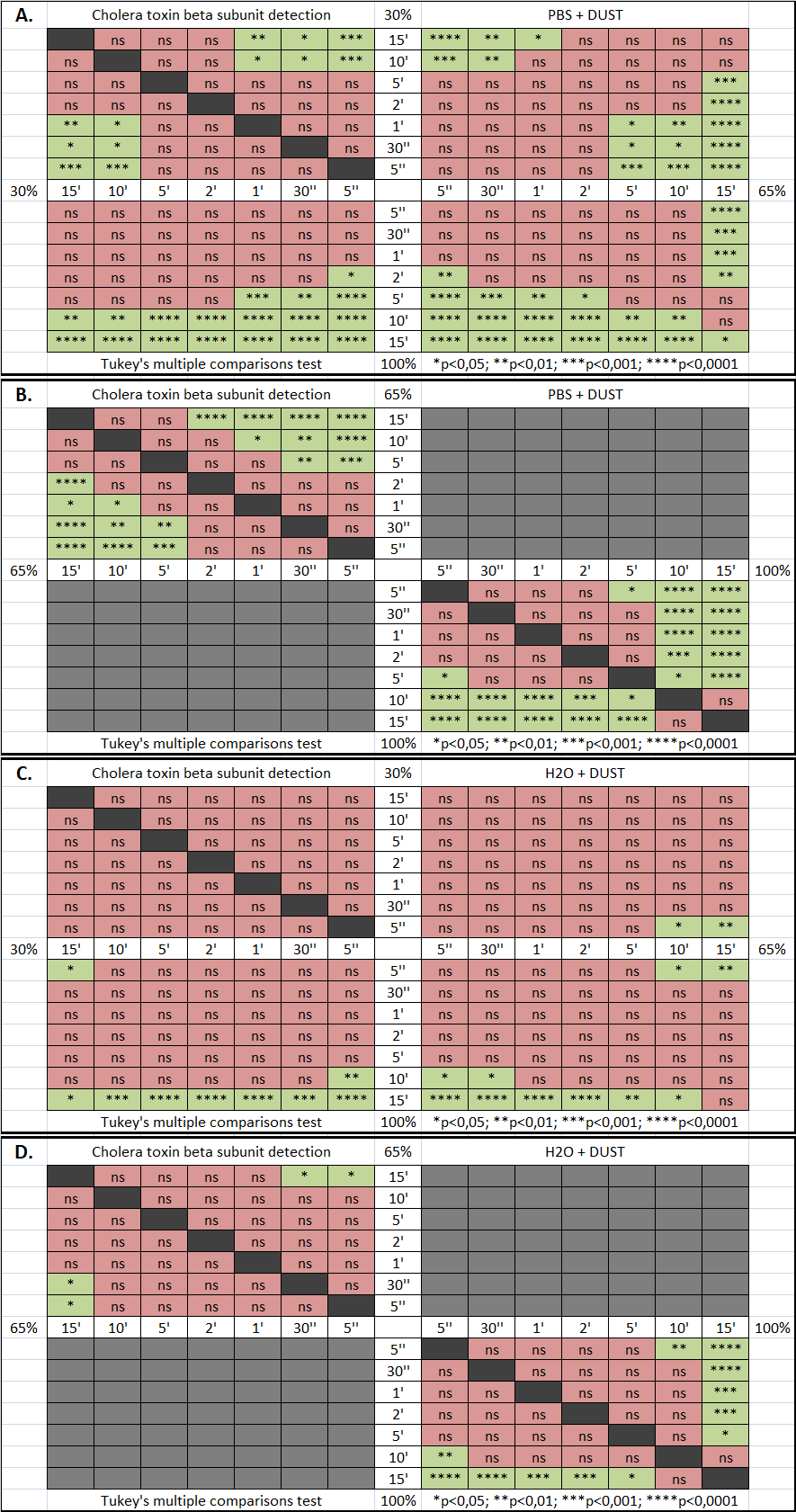

Supplement: FIGURE S10 — Statistical analysis between different points of cholera toxin beta subunit detection after sonication. Results of multiple comparisons of CTB detection every point with every point using the Tukey criterion are shown. On (A,B) plots results after different duration and power of ultrasonication in PBS-DUST matrix are shown. Same condition points, but in H20-DUST are plotted on (C,D) plots. Statistical significance is: ∗p < 0.05; ∗∗p < 0.01; ∗∗∗p < 0.001; ∗∗∗∗p < 0.0001. [file Image_10.TIF]
